# Elevated tropospheric ozone and crop production: potential negative effects and plant defense mechanisms

**DOI:** 10.3389/fpls.2023.1244515

**Published:** 2024-01-09

**Authors:** Farzana Nowroz, Mirza Hasanuzzaman, Ayesha Siddika, Khursheda Parvin, Pedro Garcia Caparros, Kamrun Nahar, P.V. Vara Prasad

**Affiliations:** ^1^ Department of Agronomy, Sher-e-Bangla Agricultural University, Dhaka, Bangladesh; ^2^ Department of Horticulture, Sher-e-Bangla Agricultural University, Dhaka, Bangladesh; ^3^ Agronomy Department of Superior School Engineering, University of Almería, Almería, Spain; ^4^ Department of Agricultural Botany, Sher-e-Bangla Agricultural University, Dhaka, Bangladesh; ^5^ Department of Agronomy, Kansas State University, Manhattan, KS, United States

**Keywords:** abiotic stress, antioxidants, atmospheric pollutants, oxidative stress, photosynthesis, prooxidant, reactive oxygen species

## Abstract

Ozone (O_3_) levels on Earth are increasing because of anthropogenic activities and natural processes. Ozone enters plants through the leaves, leading to the overgeneration of reactive oxygen species (ROS) in the mesophyll and guard cell walls. ROS can damage chloroplast ultrastructure and block photosynthetic electron transport. Ozone can lead to stomatal closure and alter stomatal conductance, thereby hindering carbon dioxide (CO_2_) fixation. Ozone-induced leaf chlorosis is common. All of these factors lead to a reduction in photosynthesis under O_3_ stress. Long-term exposure to high concentrations of O_3_ disrupts plant physiological processes, including water and nutrient uptake, respiration, and translocation of assimilates and metabolites. As a result, plant growth and reproductive performance are negatively affected. Thus, reduction in crop yield and deterioration of crop quality are the greatest effects of O_3_ stress on plants. Increased rates of hydrogen peroxide accumulation, lipid peroxidation, and ion leakage are the common indicators of oxidative damage in plants exposed to O_3_ stress. Ozone disrupts the antioxidant defense system of plants by disturbing enzymatic activity and non-enzymatic antioxidant content. Improving photosynthetic pathways, various physiological processes, antioxidant defense, and phytohormone regulation, which can be achieved through various approaches, have been reported as vital strategies for improving O_3_ stress tolerance in plants. In plants, O_3_ stress can be mitigated in several ways. However, improvements in crop management practices, CO_2_ fertilization, using chemical elicitors, nutrient management, and the selection of tolerant crop varieties have been documented to mitigate O_3_ stress in different plant species. In this review, the responses of O_3_-exposed plants are summarized, and different mitigation strategies to decrease O_3_ stress-induced damage and crop losses are discussed. Further research should be conducted to determine methods to mitigate crop loss, enhance plant antioxidant defenses, modify physiological characteristics, and apply protectants.

## 1 Introduction

Ozone (O_3_) is a major environmental stressor that affects crop production negatively. The concentration of O_3_ is predicted to increase by 25% by 2050 and 60% by 2100 (Meehl et al., 2007; Jimenez-Montenegro et al., 2021). Ozone is present both in the stratosphere and the troposphere, where the stratospheric O_3_ is known as the “good O_3_” or “O_3_ layer” because it absorbs the harmful ultraviolet (UV-B) rays of sunlight and acts as a UV-ray filter, preventing its harmful effects on living organisms on Earth including plants. Tropospheric or ground-level O_3_ is responsible for promoting damaging effects on living cells, organs, and species; therefore, it is known as “bad O_3_” (Xu, 2021). Tropospheric O_3_, namely ground-level O_3_, is an air pollutant and secondary air pollutant recommended by air quality guidelines in all countries. Although they are generated in the troposphere by sunlight-driven chemical processes that combine nitrogen oxides (NOx) and volatile organic carbons (VOCs), the use of fossil fuels by the industrial and transport sectors has significantly increased the level of O_3_ in the troposphere, mainly due to the release upon combustion of several O_3_ precursors (Zhang et al., 2019; Hasan et al., 2021). Consequently, the phytotoxic effects of increased tropospheric O_3_ on many plant species, losses in global crop production, especially in industrial and urban areas, and damage to ecological health and environmental sustainability in the long term are well known (Monks et al., 2015; Ramya et al., 2023). However, the generation of O_3_ occurs in the troposphere through photochemical reactions between precursors emitted by anthropogenic, natural, and agronomic sources, such as NOx, carbon monoxide, methane, VOCs, and peroxyacetyl nitrate (Vainonen and Kangasjärvi, 2015).

Plants take up O_3_ through the stomata, and it is later converted into reactive oxygen species (ROS) in the apoplast. The accumulation of ROS damages the photosynthetic machinery, causes stomatal closure, and degrades ribulose-1,5-bis-phosphate carboxylase/oxygenase (RuBisCO) (Ren, 2021). However, the damage caused by O_3_ to plants depends on the dose and exposure time. Ozone exposure can be categorized as acute or chronic. High doses of O_3_ over short periods (acute damage) may lead to programmed cell death and leaf damage, particularly in sensitive plant species. However, a lower dose of O_3_ for a longer duration (chronic damage) affects the photosynthetic rate, causing growth reduction and rapid leaf senescence, with or without visible damage to the leaves (Chen et al., 2018; Emberson et al., 2018). Increased O_3_ exposure results in higher yield loss through foliar damage, suppression of photosynthesis with altered carbon translocation, and consequently earlier plant senescence occurs. Numerous distinct alterations in gene expression, metabolic profiles, and enzyme activity occur in plants exposed to O_3_. Acute O_3_ exposure in sensitive accessions led to increased cell death, lesion formation, and reduced photosynthesis (Morales et al., 2021). Owing to its high reactivity and instability, O_3_ can cause oxidative stress in apoplast plant cells by chemically altering different components such as proteins to form short-lived ROS. Plants activate antioxidant defense mechanisms to scavenge ROS and prevent their negative effects. Ozone-tolerant plants have different characteristics that suppress cellular toxic elements, such as peroxidation, and maintain cell membrane stability, which is made possible by the activation of both enzymatic and non-enzymatic antioxidant components (Dhevagi et al., 2021; Ramya et al., 2021a). Thus, plants can become tolerant to O_3_, which ultimately safeguards their yield under adverse circumstances.

The generation of O_3_ has a strong relationship with different meteorological factors, being positively correlated with sunshine hours and negatively correlated with wind speed and relative humidity (Markovic and Markovic, 2005). Consequently, the level of O_3_ changes over the season and even during the day, with high values in the day and dry months and low values in the night and wet months (Jain et al., 2005). It has been reported that the diurnal variation in O_3_ coincides with the intensity of solar radiation and higher air temperatures. Nevertheless, ground-level O_3_ concentrations remain low under nighttime conditions because there is no photolysis of nitrogen dioxide (NO_2_) or photooxidation of O_3_ precursors (Ma et al., 2021). Upon exposure to ultraviolet (UV) radiation, NO_2_ dissociated into nitric oxide (NO) and oxygen (O). The short lifetime of O_3_ is related to its ability to react with NO to produce NO_2_ and O_2_ again (Simon et al., 2014).

In this review, we explored the processes involved in O_3_ uptake by plants and their perception of this pollutant. In this study, we investigated the multivariate effects of O_3_ on plant growth, nutrition, physiology, yield, and oxidative stress. In addition, we discuss strategies for mitigating the phytotoxic effects of O_3_ and enhancing the performance of O_3_-affected plants. These strategies include crop management practices, carbon dioxide (CO_2_) fertilization, using chemical elicitors, proper nutrient management, and the selection of tolerant crop varieties. Therefore, this review aims to provide a comprehensive understanding of O_3_-induced damage in plants and techniques for improving O_3_ tolerance, thereby shedding light on O_3_-related research.

## 2 Ozone uptake by plants

Ozone pollution has been perceived mostly as a daytime problem because gas generation occurs through complex photolytic reactions, and the leaf stomata are open during this period, allowing O_3_ uptake into plants. Nevertheless, O_3_ uptake may also occur at night because the stomata are not completely closed (Dawson et al., 2007; Rannik et al., 2009). Furthermore, O_3_ can enter the leaves by direct absorption through the leaf exterior surfaces, albeit less than the amount of O_3_ entering the stomata (Pleijel et al., 2004). Besides the uptake of O_3_ via the stomata, O_3_ can also be deposited onto agricultural systems via non-stomatal pathways (e.g., soil and cuticular deposition) (Morales et al., 2021). Stomata are the first barrier to overcome the damage in plants caused by O_3_ because stomatal aperture control is responsible for O_3_ flux into the leaves (Fiscus et al., 2005). The size of the stomatal aperture is controlled by the activity of the guard cell ion channels and transporters responsible for the movement of osmolytes across the tonoplast and plasma membrane (Kollist et al., 2011; Hedrich, 2012). Ion channel regulation is controlled by reversible protein phosphorylation by protein kinases and phosphatases (Evans et al., 2005).

Three levels of O_3_ concentration have been established: low (20–70 nL O_3_ L^-1^), moderate (70–150 nL O_3_ L^-1^), and high (>150 nL O_3_ L^-1^) (Grulke and Heath, 2020). According to European Environmental Legislation, the threshold O_3_ concentration should not exceed 40 ppb to protect crops during the growing season (Proietti et al., 2021). The chronological and spatial sequences of these actions define how O_3_ exposure affects plant physiology. First, O_3_ enters the leaf apoplast via the stomata, where it is degraded into secondary ROS at two different locations (mesophyll and guard cell walls) by triggering specific calcium (Ca) signatures in the cytosol. High concentrations of Ca and ROS in the cytosol of guard cells result in stomatal closure and, consequently restriction of O_3_. There is a high influx of O_3_ in the apoplast of mesophyll cells, inducing excessive ROS accumulation that is not scavenged by apoplastic antioxidants, such as ascorbate (AsA) (Grulke and Heath, 2020). Higher accumulation of ROS in the apoplast triggers several downstream signaling pathways that work in parallel, in series, or both (Roychowdhury et al., 2019; Li et al., 2023). Reactive oxygen species signaling and apoplast propagation in the apoplast are related to the induction of respiratory burst oxidase homolog (RBOH) activity and type III peroxidases. The signaling process in the apoplast is then quickly relayed to the chloroplast, where the ROS signal is amplified by chloroplastic ROS formation. This process is regulated by heterotrimeric G proteins (Joo et al., 2005; Booker et al., 2012). The intracellular pathways promoted by ROS involve the activation of mitogen-activated protein kinase (MAPK) cascades, modification of intracellular redox homeostasis, and generation of NO (Morales et al., 2021).

The absence of α- or β-subunits of the G-protein in *gpa1* or *agb1* mutants resulted in the first early peak of ROS not being generated, while only the G α-subunit was required for the second peak of ROS accumulation (Joo et al., 2005; Vainonen and Kangasjärvi, 2015). These results indicate that ROS synthesis in the chloroplasts may be involved in apoplast signaling process (Shapiguzov et al., 2012; Sierla et al., 2013). Retrograde signaling from the chloroplasts to the nucleus may cause modifications in nuclear gene expression (Leister, 2012; Estavillo et al., 2013). This signaling process may be achieved via ABI4 (Koussevitzky et al., 2007), WHIRLY 1 (Isemer et al., 2012), and PTM (PHD-type transcription factor with transmembrane domains) transcription factors (Sun et al., 2011) as well as metabolites such as Mg-protoporphyrin IX (Strand et al., 2003), heme (Woodson et al., 2011), 3′-phosphoadenosine-5′-phosphate (PAP) (Xiao et al., 2012) and β-cyclocitral (Ramel et al., 2012).

## 3 Plant sensing and indication of O_3_ stress

Plants respond differentially to ozone exposure. The response pattern of ozone-tolerant and sensitive plants has been reported in several studies. Ozone-responsive proteins and signaling molecules are primarily involved in ozone sensing. The mechanisms involved in O_3_ sensing are as follows: a) recognition by an apoplastic receptor protein, which can be changed by the ROS synthesis related to O_3_ breakdown, b) oxidation of plasma membrane lipids resulting in the generation of lipid-based signaling molecules that are further sensed, and c) modification in the redox homeostasis due to the participation of ascorbic acid (AsA), glutathione (GSH) or the ratio NAD(P)H/NAD(P) (nicotinamide adenine dinucleotide phosphate-with and without hydrolase) (Kangasjärvi et al., 2005).

Excess ROS production due to O_3_ exposure causes cellular damage; however, ROS initially act as signaling molecules (Jaspers and Kangasjärvi, 2010). ROS can alter signal transduction proteins within membranes in response to O_3_ stress (Rossard et al., 2006). Consequently, several cellular changes occur, such as a) depolarization and dysfunction of the membrane, b) modification of cell wall compounds, c) promotion of MAP kinase protein cascades to generate new proteins through transcription factor activation, d) ozonolysis of double bonds in the unsaturated fatty acids of cell membranes, and e) lipid peroxidation in the membrane (Evans et al., 2005; Iriti and Faoro, 2008; Sharma et al., 2012; Emberson et al., 2018).

Baier et al. (2005) noted that the signaling process of O_3_ from the chemical reaction sites in the apoplast or plasma membrane to the cytosol can be associated with O_3_ induced ROS production, particularly H_2_O_2_, which functions as a diffusible messenger (Laloi et al., 2004) and modulates cytosolic AsA and GSH (Gomez et al., 2004). Extracellular peroxidases (PRX) and plasma membrane-bound NADPH oxidases (RBOH) enhance ROS generation under O_3_ stress (Kangasjärvi et al., 2005). In addition to H_2_O_2_, ethylene and salicylic acid (SA) have been reported as secondary or tertiary messenger molecules involved in O_3_ sensing, because O_3_ or O_3_-derived ROS can activate them. Both ethylene and SA can increase oxidative signaling (Sandermann, 2000; Kangasjärvi et al., 2005).

Exposure of plants to O_3_ also triggers SA-induced cell death because SA can inhibit the main H_2_O_2_-scavenging enzymes such as catalase (CAT) and ascorbate peroxidase (APX) (Faoro and Iriti, 2009). Under severe exposure to O_3_, NO synthesis increased. This molecule plays a crucial role in the signaling of plants subjected to stress conditions. Moreover, NO can react with H_2_O_2_ and NO donors such as sodium nitroprusside (SNP), leading to the accumulation of H_2_O_2_ in plants (Astier et al., 2018). Another consequence of long-term exposure to O_3_ is increased cell wall lignification in many plants, which helps decrease O_3_ penetration (Cabané et al., 2012).

The physiological damage caused by O_3_ exposure is related to photosynthetic reduction, ROS generation, increased dark respiration, and reduced crop yield (Cailleret et al., 2018). More accurately, the main effects of chronic exposure to O_3_ are a reduction in the photosynthetic rate, growth reduction, and premature senescence without visible symptoms (Krupa, 2003). In contrast, acute exposure to O_3_ results in cell death and other adverse effects (Kangasjärvi et al., 2005). Symptoms of O_3_ exposure can be observed between the veins on the adaxial leaf surfaces of older and middle-aged leaves; however, symptoms can appear on both leaf surfaces (adaxial and abaxial) in some species if the damage is severe (Cho et al., 2011; Vaultier and Jolivet, 2015). Induced chlorosis and bronzing (several spots on the leaves) are the most frequent symptoms of chronic O_3_ exposure, and acute exposure may result in a higher number of visible lesions (Kangasjärvi et al., 2005).

## 4 Ozone as a Prooxidant and O_3_-induced oxidative stress

**Figure 1:**
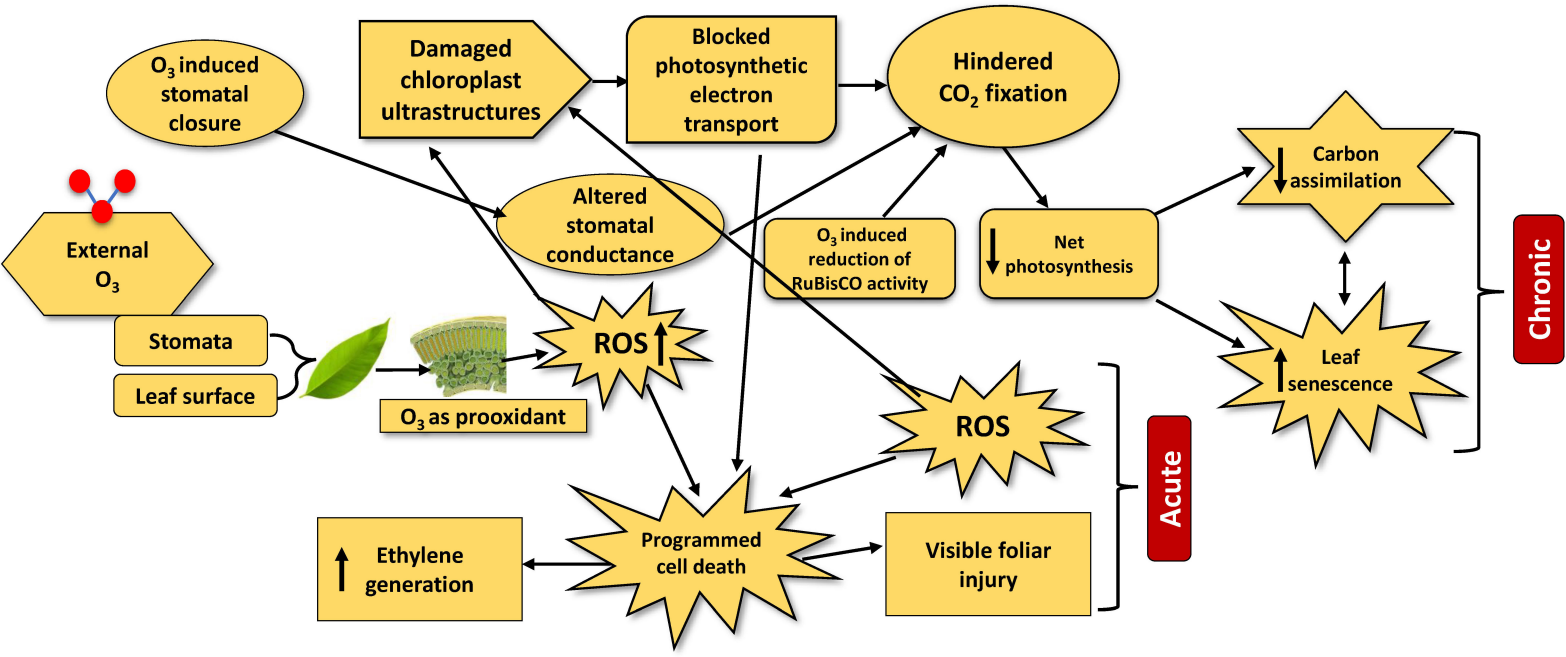
The major physiological processes and mechanisms involved in leaf injury under chronic and acute ozone exposure. The upward arrow inside boxes indicates increase and downward arrow indicates decrease.

Ozone can directly generate ROS in the leaf mesophyll and guard cell walls after entering the stomata (Grulke and Heath, 2020). Higher exposure activates the pro-oxidant activity of O_3_ by increasing ROS accumulation and decreasing the antioxidant machinery in plant cells (Dhevagi et al., 2021; Ramya et al., 2021a). Moreover, the overgeneration of ROS and the reaction between O_3_ and plasma membrane lipids results in peak levels of superoxide and thiobarbituric acid (Marchica et al., 2019). This phenomenon confirmed the oxidative stress in plant cells upon O_3_ exposure. Under O_3_ exposure, plants suffer several physiological damages, resulting in a lower photosynthetic rate, which in turn results in growth reduction, premature senescence, and cell death, mainly due to the generation of ROS. To counteract these harmful effects, plants activate different antioxidant systems to scavenge the reactive molecules (**Figure 1**).

Several studies have investigated the pro-oxidant effects of O_3_ and the respective changes in redox homeostasis. Rice (*Oryza sativa* L.) seedlings of three different cultivars, Nipponbare and BRRI dhan28 (sensitive to O_3_), and L81 (O_3_-tolerant introgression line), were grown from April to October 2016 in a controlled climate greenhouse. Plants were sprayed with 80 ppb O_3_ for 7 hours daily, five weeks after transplantation to induce acute stress. Owing to tolerance differences between cultivars, malondialdehyde (MDA) content increased in sensitive cultivars but not in the tolerant line. However, the total leaf AsA did not show any significant differences among the cultivars (Ashrafuzzaman et al., 2017). Tobacco (*Nicotiana tabacum* L.) plants exposed to 300 nmol mol^-1^ for 4 h at midday showed clear symptoms of leaf necrosis and a reduced net photosynthetic rate. Oxidative stress caused by exposure to O_3_ results in an increase in H_2_O_2_ and MDA contents, and ion leakage (Guo et al., 2009). In another experiment, two cultivars of bean (*Phaseolus vulgaris* L.) with different O_3_ tolerance levels (O_3_-sensitive “Cannellino” and O_3_-tolerant “Top Crop”) were subjected to a severe O_3_ stress (165 nL L^-1^). Biochemical characterization of different cultivars showed that exposure to this phytotoxic air pollutant increased superoxide dismutase (SOD) and CAT activities in both cultivars, which was more pronounced in the tolerant genotype (Guidi et al., 2010). Similarly, two cultivars of soybean (*Glycine max* L.) with opposed degrees of O_3_ tolerance (O_3_-sensitive “Mandarin (Ottawa)” and O_3_-tolerant “Fiskeby III”) were treated with 70 ppb for 4 days (7 h day^-1^). Histochemical assays performed on these cultivars showed an accumulation of H_2_O_2_ via 3,3′-diaminobenzidine (DAB) in the sensitive cultivar, whereas no spots were detected in the tolerant cultivar. Nevertheless, superoxide anion (O_2_^•-^) generation showed different trends in the two cultivars when tested by nitro blue tetrazolium chloride (NBT) reduction. There were no significant differences in SOD and glutathione reductase (GR) activities between the genotypes or O_3_ treatments (Chutteang et al., 2016). Similarly, Szpunar-Krok et al. (2020) tested the effects of two doses of O_3_ (5 and 10 ppm), different exposure times (2, 4, 8, 12, and 16 min), and two application periods (21 and 28 d after sowing) on potatoes (*Solanum tuberosum* L.). This experiment revealed a significant decrease in the total antioxidant activity based on ABTS^•+^ and DPPH^•^ radical assays. **Table 1** includes additional information on studies that investigated the pro-oxidant effects of O_3_ on different crops.

**Table 1:** Effects of ozone stress in crops and the different defense responses triggered in each species.

Plant species	O_3_ levels	Stress period	Physiological and defense response		References
*Brassica oleracea* (L.)	70 ppb	3 days	CAT, APX, and POD activities	Enhanced	Rozpądek et al. (2013)
*Capsicum baccatum* (L.)	Average of 0.172 ppb	62 days (6 h day^-1^)	Lipid peroxidation and protein carboxylation in leaf	Increased	Bortolin et al. (2014)
			SOD, CAT and APX activities	Decreased	
*Glycine max* (L.)	200 ppb	4 h	Leaf AsA content	Increased	Gillespie et al. (2011)
			DHAR activity	Increased	
			GR activity	Reduced	
			MDHAR, APX, SOD and CAT activities	No change	
*Oryza sativa* (L.)	150 ppb	6 h	Production of O_2_^.-^ and lipid peroxidation in leaf	Increased	Ueda et al. (2013)
*O. sativa* (L.)	51 ppb	30 days (7 h day^-1^)	MDA and proline contents	Increased	Ramya et al. (2021b)
*Solanum tuberosum* (L.)	Average of 50 ppb	60 days (6 h day^-1^)	MDA and H_2_O_2_ content; SOD, GR and APX activities	Increased	Kumari et al. (2015)
*Triticum aestivum* (L.)	80 ppb	30 days (8 h day^-1^)	Electrolyte leakage, lipid peroxidation, POD, and CAT activities	Increased	Zheng et al. (2011)
*T. aestivum* (L.)	Average of 66 ppb	5 months (8 h day^-1^)	Lipid peroxidation, MDA, H_2_O_2_, O_2_ and OH^.-^ content in leaves	Enhanced	Ghosh et al. (2020b)
*Vigna unguiculata* (L.)	40, 50, 60, 70 and 80 ppb	15 min, twice a day	AsA activity, proline content	Increased	Malaiyandi and Natarajan (2014)

(AsA, Ascorbate; DHAR, Dehydroascorbate reductase; GR, Glutathione reductase; MDHAR, Monodehydroascorbate reductase; APX, Ascorbate peroxidase; SOD, Superoxide dismutase; CAT, Catalase; O_2_^.-^, Superoxide anion; H_2_O_2_, Hydrogen peroxide; GR, Glutathione reductase; O_2_, Oxygen; OH^.-^, Hydroxyl radical).

## 5 Plant responses to O_3_

**Figure 2:**
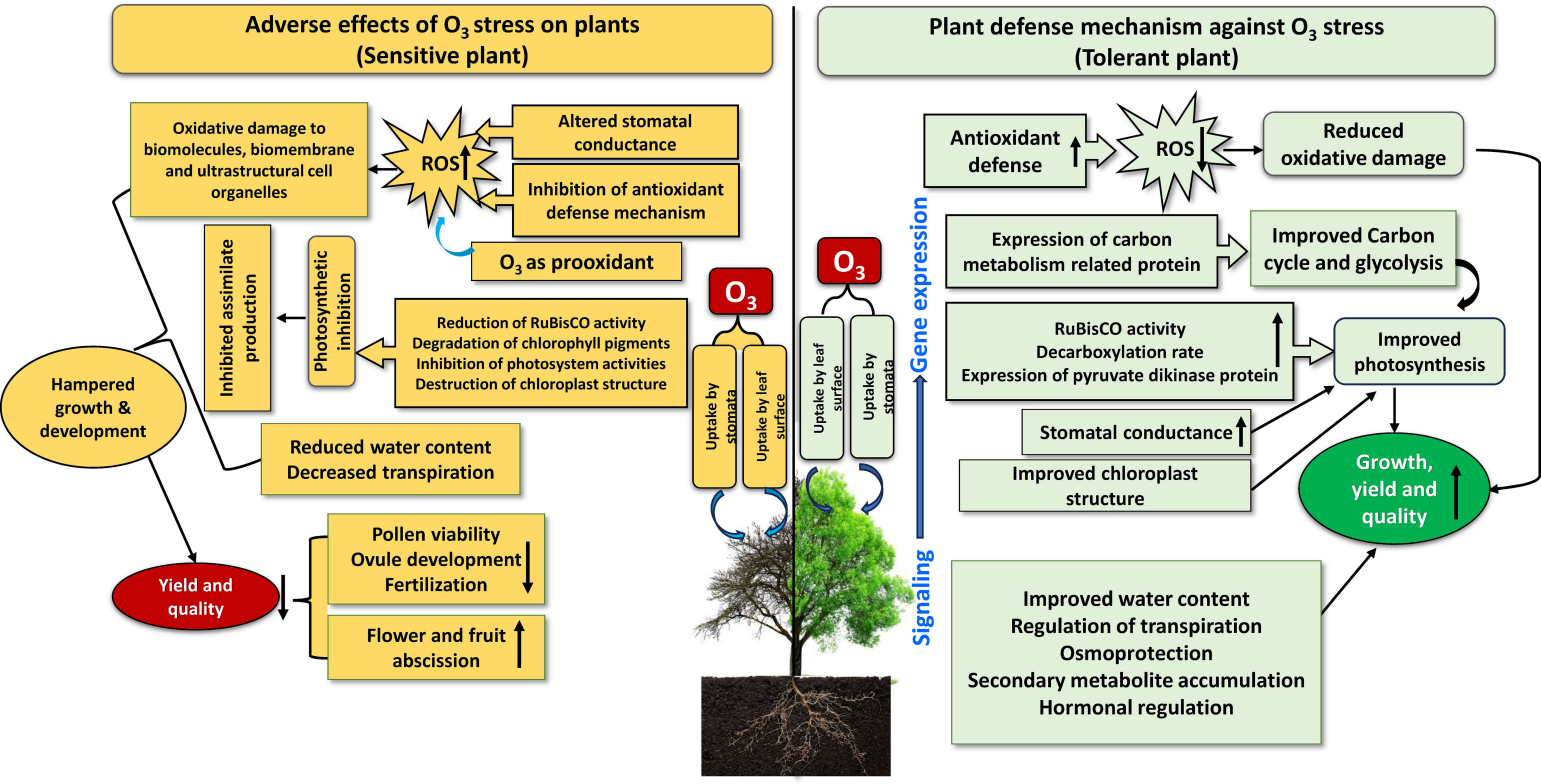
A schematic illustration of the response of sensitive and tolerant plants to ozone. The upward arrow inside boxes indicates increase and downward arrow indicates decrease.

The response of plants to O_3_ stress depends on the concentration and duration of exposure. Ozone may also be deposited in plant cells by non-stomatal channels, such as soil and cuticular deposits, in addition to being taken up by the stomata (Morales et al., 2021). Because stomatal opening and closing regulate the flow of O_3_ into the leaves, stomata act as an initial defense against O_3_ damage in plants. However, the activities of transporters and ion channels in guard cells that move osmolytes through the tonoplast and plasma membrane regulate the size of the stomatal aperture. After accumulation in plant cells, O_3_ induces short-term responses in plants depending on the frequency of stress exposure, such as the production of noticeable fine bronze or pale-yellow spots on the upper surface of the leaves. However, O_3_-induced phytotoxicity begins when O_3_ diffuses into the vacuolar space of the leaves through stomatal openings, promoting oxidative stress by encouraging the rapid production of ROS in the apoplast (**Figure 2**). Additionally, O_3_ can directly diffuse into the cytosol through the cell membrane, generating ROS that alters stomatal conductance (*g_s_*) (Ainsworth, 2017. ) Numerous studies have used external O_3_ to investigate the responses of plants to stress in terms of growth, biomass, reproduction, and yield (**Figure 2**).

### 5.1 Crop growth

The response of plants to O_3_ varies depending on species, cultivar, and developmental stage (**Table 2**). A few varieties of wheat (*Triticum aestivum* L.) and rice show visible signs of foliage injury during their early growth stages (Ramya et al., 2021b). Ozone causes early leaf senescence and abscission, which can affect biomass and growth by allocating carbon to edible plant portions. Additionally, the O_3_-induced reduction in root growth is greater than the reduction in shoot growth because of impaired carbohydrate partitioning in the roots (Witting et al., 2009). However, it adversely affects shoot growth. Yadav et al. (2021) observed significant effects on plant growth when experimenting with four Indian wheat cultivars (early-sown cultivars HUW468 and HD3086; late-sown cultivars HUW234 and HD3118). They reported a greater reduction (26%) in the aboveground biomass of early-sown cultivars than that of late-sown cultivars (21%) under ambient and elevated O_3_ (ambient+20 ppb).

**Table 2:** Overview of research on O_3_ responses in crop growth of different plant species.

Plant species	O_3_ levels	Observed parameters and effects		References
*Brassica chinensis* (L.)	150 ppb O_3_ for 4 h day^-1^	Crop dry matter	Reduced by 7%	Han et al. (2020)
*Oryza sativa* (L.)	50 ppb	Chlorophyll content	Reduced by 17-35%	Ramya et al. (2021b)
*Pisum sativum* (L.)	151.2 ppb	Reduced vegetative growth and increased visible foliar injury		Yendrek et al. (2015)
*Triticum aestivum* (L.)	Average charcoal filtered O_3_ (13.3 ppb) and average non-filtered O_3_ (34.6 ppb)	Total above-ground biomass	Reduced by 5%	Pleijel et al. (2018)
*T. aestivum* (L.) *cvs*. Akbar, Sufi, Bijoy, Shatabdi, BARI gom -26, Gourab, BARI gom-25, Prodip, Sourav and Kanchan	50, 60, 80, 100, 120, 135, 150, and 200 ppb for 14, 11, 8, 6, 5, 4, 3, and 1 days, respectively, for 8 h day^-1^	Visible leaf injury	Increased in all cultivars	Saitanis et al. (2014)
		Total shoot biomass	Decreased by 25% (Akbar) and 20% (Sufi)	
		Total dry weight	Reduced	
*T. aestivum* (L.) *cvs.* HUW-37 and K-9107	Elevated O_3_ at 40, 60 and 80 days after germination (DAG)	Plant height at 60 DAG	Decreased by 29% (HUW-37) and 21% (K-9107)	Mishra et al. (2013)
		Leaf number at 80 DAG	Reduced by 28.2% (HUW-37)	
		Total biomass at 80 DAG	Decreased by 49.2% (HUW-37) and 43.8% (K-9107)	
*T*. *aestivum* (L.) *cvs.* HUW 510 and Sonalika	Ambient + 10 ppb, Ambient + 20 ppb	Vegetative parts	Reduced plant height, root length, leaf number, and leaf area in both cultivars	Sarkar and Agrawal (2010)
*Vigna radiata* (L.) *cvs.* HUM-1, HUM-2, HUM-6, HUM-23, HUM-24 and HUM-26	Ambient and elevated O_3_ (ambient + 10 ppb O_3_)	Plant height at 40 DAG	Decreased by 26% (HUM-1) and 10% (HUM-23)	Chaudhary et al. (2013)
		Number of leaves plant^-1^ at 40 DAG	Reduced by 24% (HUM-1), 17% (HUM-2),12% (HUM-6), 9% (HUM-26) and 8% (HUM-24)	
*V. unguiculata* (L.) *cvs*. Blackeye and Asontem	39 ppb, 24 h mean	Leaf area per plant^-1^, specific leaf area, whole-plant dry masses, and root–shoot ratio were decreased		Tetteh et al. (2015)

### 5.2 Plant physiology and metabolism

Ozone-induced phytotoxicity negatively affects plant physiology and metabolism, including photosynthesis, respiration, transpiration, relative water content, and secondary metabolite accumulation in various crop plants (Cho et al., 2011; Ainsworth et al., 2012; Hassan et al., 2017). In particular, evaluating physiological processes is a more accurate method for assessing intrinsic O_3_-induced injuries in plants because physiological damage can start at lower O_3_ concentrations and before the onset of visible impairment (Pandey et al., 2019). Several studies have evaluated the detrimental effects of ambient and elevated O_3_ on the physiological processes in plants (**Table 3**). O_3_-induced injuries are also related to reduced dry mass accumulation in leaves, lower leaf area-based antioxidant levels, and altered *g_s_*. However, the O_3_ uptake is linked to *g_s,_* which varies according to the absorption capacity of the cuticle and stem in different plant species, such as the cuticle and stem. For example, increasing the amount of O_3_ leads to a reduction in *g_s_* and stomatal pore area in tomatoes (*Solanum lycopersicum* L.) (Thwe et al., 2014). Moreover, Yendrek et al. (2015) reported that legume crops, such as peas (*Pisum sativum* L.), soybeans, and beans, display reduced net photosynthetic rates and leaf longevity. Cabbage (*Brassica oleracea* var. *capitata*) (cv. Tekila and Primero) plants showed decreased photosynthetic rates (71.2%), stomatal conductance (81.03%), and chlorophyll content (32.98%) (Ramakrishnan et al., 2023) when exposed to O_3_ (200 ppb). Ozone-induced stomatal closure occurs because of the inhibition of carbon assimilation in chloroplasts, which leads to an accelerated internal carbon dioxide (CO_2_) concentration (Ainsworth et al., 2012). Moreover, O_3_ exposure directly impacted the net CO_2_ assimilation rate and CO_2_ fixation ability negatively. Total carbon sequestration and transpiration rates were also related to reduced *g_s_* and photosynthetic rates. It has also been observed that O_3_-induced alterations in the CO_2_ assimilation rate further influence plant respiration, leading to reduced crop growth and productivity (Ainsworth, 2017). In addition to ROS production, the concentration of secondary metabolites in plant cells is altered by O_3_-induced stress. Higher concentrations of O_3_ activate the first enzyme of the phenylpropanoid pathway, leading to a higher accumulation of flavonoids, phenolic acids, monolignols, GSH, gamma-aminobutyric acid (GABA), terpenoids, and volatile organic compounds such as isopropanoids (Mikkelsen et al., 2015).

**Table 3:** Ozone-induced responses in crop physiology and metabolism of different plant species.

Plant species	O_3_ levels	Observed parameters and effects		References
*Capsicum baccatum* (L.)	Mean O_3_ concentration of 171.6 ppb for 62 days	Secondary metabolite profile, e.g., total phenolic compounds	Increased by 17% in pericarp	Bortolin et al. (2016)
		Total antioxidant potential	Decreased by 87% in seeds	
*Glycine max* (L.)	Elevated O_3_ (ambient + 40 ppb O_3_)	Photosynthetic pigment contents at both flowering and seed filling stages	Reduced	Zhang et al. (2014)
		Net photosynthetic rate at both flowering and seed filling stages	Diminished	
		Chlorophyll *a* fluorescence rate at both flowering and seed-filling stages	Reduced	
*Hordeum vulgare* (L.)	0 ppb and 100 ppb O_3_	Accumulation of twenty-five secondary metabolites, including phenylpropanoids, phenolamides and hydroxynitrile glucosides	Altered	Mikkelsen et al. (2015)
*Nicotiana tabacum* (L.)	Ambient O_3_	Epicuticular wax on leaves and stomatal aperture movement	Damaged and reduced	Alves et al. (2016)
*Solanum lycopersicum* (L.)	0.5 ppb of O_3_	PS II activities	Hampered	Thwe et al. (2014)
*Triticum aestivum* (L.)	50, 60, 80, 100, 120, 135, 150, and 200 ppb for 14, 11, 8, 6, 5, 4, 3, and 1 days, respectively, for 8 h day^−1^	Total chlorophyll	Reduced	Saitanis et al. (2014)
		Leaf greenness	Decreased	
		Carotenoid content	Reduced	
*T. aestivum* (L.)	30 and 80 ppb; 4 weeks	Stomatal conductance (*g_s_*)	Reduced	Harmens et al. (2019)
		Light-saturated photosynthesis	Reduced	
		Chlorophyll content index	Decreased	
*Vigna unguiculata* (L.)	39 ppb, 24 h mean	*g_s_*	Reduced	Tetteh et al. (2015)

Ozone exposure also causes a nutrient imbalance in plant cells by altering the allocation of nutritional elements and their ratios in the belowground (root) and aboveground parts (stem and leaves) and disrupting other physiological activities. For example, the potassium (K), calcium (Ca), sodium (Na), iron (Fe), and zinc (Zn) contents in potato tubers are lower under elevated O_3_ than under ambient conditions, which lowers tuber quality (Kumari and Agrawal, 2014). Similarly, Ghosh et al. (2020a) revealed that the concentrations of nitrogen (N), phosphorus (P), K, magnesium (Mg), and Ca were reduced in the leaves and shoots of wheat under ambient and elevated (ambient+20 ppb) O_3_ stress from 2 weeks after germination to maturity for 4 h. However, carbon was enhanced under the same stressful conditions, which led to an increase in the C:N and C:K ratios in the leaves. Similarly, as O_3_ alters the absorption and distribution of macronutrients owing to changes in organic matter mineralization, the uptake of other nutrients is also influenced. Under O_3_ stress, copper (Cu) concentration was significantly reduced in the leaves, shoots, and roots, although the reduction was higher in the shoots than in the roots. Ozone-induced reduction in leaf N has also been observed in previous studies (Chen et al., 2015; Pandey et al., 2018).

### 5.3 Reproductive development

Reproductive development is the key determinant of plant productivity and species distribution. Increased O_3_ has a detrimental effect on the reproductive system, primarily because it alters the allocation of carbon among tissues and directly affects plant reproductive processes (Gillespie et al., 2015). Several studies have demonstrated the effects of O_3_ on flower initiation, floral development, pod formation, seed quality, seedling germination, and seedling vigor have been demonstrated in several studies. For example, Gillespie et al. (2015) demonstrated that O_3_ adversely affected pollen viability, pollen germination, germ tube growth, pollen-stigma interactions, and fertilization in tomatoes, leading to decreased seed size, weight, and quality. Consequently, O_3_-induced lower pollen viability or ovule development leads to reduced fertilization. Moreover, elevated O_3_ levels promoted flower and pod abscission. For example, decreased pod production without an effect on flower production was observed in soybeans when plants were exposed to 150 ppb of O_3_ inside O_3_ chambers. Fruit number, fruit size, seed number, and seed size also decrease at this level of O_3_ stress in soybeans (Leisner et al., 2014). Additionally, research suggests that the effects of O_3_ exposure vary depending on the stage of plant growth, affecting flowering patterns in various ways and affecting the pollination and reproduction of annual crops and wild species. According to a recent investigation by Duque et al. (2021a), wild mustard (*Sinapis arvensis* L.) plants exposed to 120 ppb O_3_ for 6 h day^-1^ at earlier stages had more open flowers than the corresponding controls, whereas plants exposed to the same concentrations of O_3_ at later stages tended to have fewer open flowers. Similarly, when *S. arvensis* plants were exposed to 120 ppb O_3_ for 6 h day^−1^ during the flower initiation stage, flowering accelerated, increasing the proportion of open flowers in O_3_-exposed plants at the start of the flowering phase (Duque et al., 2021b). Furthermore, the O_3_-induced reduction in the photosynthesis rate inhibits the accumulation of carbohydrates in pollen in some plant species, conferring adverse effects on pollen germination (Zhang et al., 2017). O_3_-induced reductions in the number, size, weight, and quality of grains in cereal crops such as rice and wheat have also been observed in some studies (Banerjee and Roychoudhury, 2019; Schauberger et al., 2019). Although O_3_ has been found to have detectable effects on reproductive growth, the precise sites of action and mechanisms underlying these effects remain unknown.

### 5.4 Crop yield and quality

A significant proportion of crop yield losses is caused by tropospheric O_3_, which is a transient, volatile, secondary air pollutant and a powerful phytotoxic compound (Ainsworth, 2017). According to the results of several controlled environmental and field studies, current O_3_ concentrations in the environment have been found to negatively influence the yield and quality of several crop species worldwide, according to the results of several controlled environment and field studies (**Table 4**). For example, according to McGrath et al. (2015), yield losses of 10% and 20% were observed in soybean and maize (*Zea mays* L.), respectively, in combination with dry conditions and high seasonal temperatures, and 5% and 10%, respectively, under rainfed conditions at field levels from 1980 to 2011 due to O_3_ exposure. Additionally, when two cultivars of tropical maize were exposed to two different doses of O_3_ (ambient+15 ppb and ambient+30 ppb), a reduction in the test weight of kernel plant^-1^ of 6% and 10%, respectively, in DHM117, and 4% and 6%, respectively, in HQPM1 was observed (Singh et al., 2014a). Another long-term investigation conducted by Sinha et al. (2015) revealed that yield losses in rice, wheat, and maize were 21–26%, 27–41%, and 3–5%, respectively, under elevated O_3_. Additionally, according to the Intergovernmental Panel on Climate Change’s Special Report on Emission Scenarios (IPCC SRES) A2 Scenario, O_3_-induced worldwide yield losses in 2030 will range from 5 to 26% for wheat and 4 to 9% for maize (Avnery et al., 2011). Moreover, a recent study by Ghosh et al. (2020a) revealed that the grain yield was reduced by 45% in wheat cv. HD 2967 under ambient and elevated (ambient+20 ppb) O_3_ stress for 4 hours day^−1^ from 2 weeks after germination to maturity. Baqasi et al. (2018) reported a 49% decline in grain dry mass in wheat after 50 ppb O_3_ exposure. Ozone also influences the quality of crops in terms of starch, protein, nutrients, and oil content. For example, it affects grain quality by decreasing starch content and increasing the protein and nutritional contents of crops such as wheat and rice (Broberg et al., 2015; Frei, 2015).

**Table 4:** Overview of recent studies on O_3_ responses in crop yield and quality of different plant species.

Plant species	O_3_ levels	Observed parameters and effects		References
*Brassica napus* (L.)	Ambient + 10 ppb of O_3_	Grain yield	Reduced by 13% (Sanjukta) and 47% (Vardan)	Tripathi and Agrawal (2012)
*Glycine max* (L.)	Elevated O_3_ (ambient + 40 ppb O_3_)	Yield	Decreased by 40%	Zhang et al. (2014)
*G. max* (L.)	5.78 ppb and 137.7 ppb; 1 week	Seed production, seed protein content	Reduced by 10% and 12%, respectively	Biancari et al. (2021)
*Solanum tuberosum* (L.)	Ambient + 20 ppb of O_3_	Total fresh weight of tuber	Reduced by 48%	Kumari and Agrawal (2014)
*Triticum aestivum* (L.)	55.2 ppb	Number of ears plant^−1^	Reduced by 27% (HUW-37) and 20% (K-9107)	Mishra et al. (2013)
		Weight of ears plant^−1^	Decreased by 31% (K-9107)	
		Number of grains plant^−1^	Reduced by 21% (HUW-37) and 18% (K-9107)	
		Weight of grains plant^−1^	Reduced by 12% (K-9107) and 39% (HUW-37)	
*T. aestivum* (L.)	AOT40-21, 121 ppb of O_3_	Total grain weight	Reduced by 11%	Monga et al. (2015)
*T. aestivum* (L.)	80 ppb; 4 weeks	Grain yield	Decreased by 24%	Harmens et al. (2019)
		1000-grain weight	Reduced by 20%	
*Vigna radiata* (L.)	Ambient and elevated O_3_ (ambient + 10 ppb O_3_)	Yield	Reduced by 15% (HUM-1), 14% (HUM-2), 13% (HUM-6), 12% (HUM-24), 10% (HUM-26) and 9% (HUM-1)	Chaudhary et al. (2013)
*V. unguiculata* (L.)	50 ppb for 5 hr for 88 days after emergence	Number of seeds pod^-1^, 100-seed weight and yield plant^-1^	Reduced	Tetteh et al. (2015)
*Zea mays* (L.)	Ambient+15 ppb and ambient+30 ppb of O_3_	Kernel weight	Reduced by 10% (HQPM1) and 13% (DHM117)	Singh et al. (2014a)

## 6 Developing plant tolerance to O_3_

Higher O_3_ exposure causes greater yield losses through foliar damage, inhibition of photosynthesis with altered carbon translocation, and faster plant senescence (Osborne et al., 2019). There are opportunities to develop plant tolerance to O_3_ which ultimately protects the yield under stressful conditions. Breeding for stress tolerance and variety development can be time consuming and costly. Using physiological gateways such as the photosynthetic pathway, antioxidant defense mechanisms, and hormonal regulation to enhance plant tolerance to O_3_ could be a short-term option (Parankusam et al., 2019; Emberson, 2020). It is mandatory to identify the available options, followed by the most suitable option, to increase plant productivity where mitigation actions can be implemented. In this section, we present an overall discussion focusing on various approaches for developing plant tolerance to O_3_ and how O_3_ sensitivity can be lowered.

### 6.1 Improving photosynthetic pathways

Leaf health, *g_s_*, photosynthesis, and photosynthetic machinery are hampered by elevated O_3_ levels. Improving photosynthesis may be an important approach for attaining higher plant tolerance to O_3_ exposure. Ozone-mediated chlorophyll decline causes early senescence, but ethylene diurea (EDU) supplementation delays senescence in maize by increasing the chlorophyll content (Gupta et al., 2020; Poornima et al., 2022; Dhevagi et al., 2023). Increased expression of carbon metabolism-related proteins, which are part of the Calvin cycle and glycolysis, contributed to higher O_3_ tolerance by accumulating more starch, which was reflected in better biomass production in EDU-treated maize. In addition, EDU-induced elevation of RuBisCO activity also supports higher photosynthesis in combating O_3_ stress. Exogenous EDU increases the C_3_ and C_4_ photosynthesis rates by increasing the decarboxylation rate and expression of pyruvate phosphate dikinase protein, respectively, leading to higher O_3_ tolerance (Alfonso and Brüggemann, 2012). Sensitive wheat cultivars showed higher photosynthetic rates with exogenous EDU application under elevated O_3_ stress, which was correlated with EDU-induced higher chlorophyll content (Fatima et al., 2019). Therefore, improved photosynthesis and chlorophyll content may explain the higher biomass and yield, which later resulted in increased O_3_ tolerance due to EDU supplementation.

Exogenous catechin (5 mM) supplementation in rice under elevated O_3_ conditions reversed O_3_-induced damage by enhancing chlorophyll content and its precursor (Mg^2+^ content), *g_s_*, which resulted in higher grain production (Kittipornkul et al., 2020). Catechins can improve photosynthetic processes, thereby improving O_3_ tolerance. Recently, calcium acetate application was shown to increase photosystem (PS)-II efficiency and improve the yield performance of rice under O_3_ stress (Lakaew et al., 2022). There is a lack of knowledge regarding the mechanisms involved in improving the photosynthetic pathways to regulate plant growth and yield under O_3_ stress conditions. Further in-depth research is required to elucidate these mechanisms and the associated pathways.

### 6.2 Enhancing antioxidant defense

Plant antioxidant defenses must be upregulated to scavenge O_3_-induced excess ROS, thereby protecting cellular functions (Gupta et al., 2020). The inhibition of cellular component peroxidation and the maintenance of the subsequent stability of the cell membrane under O_3_-induced ROS overgeneration of ROS are distinct features of O_3_-tolerant plants, which are made possible by the activation of defense mechanisms, including both enzymatic and non-enzymatic antioxidant components (Gill and Tuteja, 2010; Noctor et al., 2014; Pellegrini et al., 2019; Hasanuzzaman et al., 2020). Similarly, Czarnocka and Karpińsky (2018) stated that insufficient responses to plant antioxidants cause oxidative damage and strengthen the defense responses of plants, which are required to develop tolerance to O_3_.

Both AsA and GSH are major antioxidants in the AsA-GSH cycle and are involved in regulating oxidative damage by scavenging ROS and maintaining cellular redox balance (Hasanuzzaman et al., 2020). Biogenic AgNPs and EDU-mediated higher antioxidant content (AsA and GSH) and enzymatic activities (SOD, CAT, APX, and GR) in wheat resulted in improved O_3_ tolerance, which was correlated with lower H_2_O_2_ and MDA accumulation (Pellegrini et al., 2019).

Fatima et al. (2018) evaluated the ROS-scavenging capability of O_3_-sensitive and tolerant wheat genotypes through their antioxidant responses. Kharchiya 65 (tolerant) displayed a maximum level of AsA, GSH, and flavonoids along with high free radical scavenging activities as well as lower ROS content than genotypes such as HD 2987 (sensitive) and PBW 502 (intermediately sensitive). At high O_3_ (ambient+30 ppb), both enzymatic and non-enzymatic antioxidant responses varied among the three cultivars. The highest SOD, peroxidase (POD), GR, and GPX activities were observed in HD 2987, whereas Kharchiya 65 and PBW 502 showed the lowest increases. The maximum APX and the lowest CAT activity was observed in HD 2987 and Kharchiya 65. Sensitive cultivars showed higher enzymatic antioxidant responses when they suffered from O_3_-induced elevated ROS levels. However, lowered SOD and POD activities were required in the tolerant cultivars, where ROS levels were lower than those in the sensitive ones. Higher free radical scavenging activities were observed in the tolerant (Kharchiya 65) cultivar than in the sensitive cultivar. Non-enzymatic antioxidant levels, such as those of AsA, GSH, and flavonoids, were also higher in the tolerant cultivar than in the sensitive cultivar, which is probably the most efficient mechanism for combating the elevated O_3_. This may be because higher O_3_ tolerance is highly associated with the genetic competence to preserve high AsA/DHA (Burkey et al., 2003). In cabbage (cv. Tekila and Primero), proline content was increased by 32.24%, ascorbic acid by 64.75%, CAT activity by 3.58%, and POD activity by 56%, which helped to reduce oxidative stress under O_3_ stress (200 ppb) (Ramakrishnan et al., 2023). Therefore, variations in antioxidant responses to counteract O_3_-induced oxidative stress are highly dependent on the crop species and the cultivars of the same crop species (Singh et al., 2014b).

Plant researchers are becoming increasingly interested in selecting appropriate techniques to increase plant tolerance by stimulating antioxidant activity. The supplementation of exogenous chemical substances to O_3_-exposed plants is one of the most efficient approaches for reducing oxidative stress and cellular damage through the enrichment of antioxidant defense systems (Qiu et al., 2019).

Ethylene diurea is widely used as an anti-ozonant to increase plant tolerance to O_3_ phytotoxicity and protect plants from damage (Manning et al., 2011). EDU-induced plant protection under O_3_ stress depends on the activation of antioxidant activity (Gupta et al., 2021). Therefore, EDU supplementation in wheat revealed an EDU-mediated active role of apoplastic SOD, CAT, and amino methyltransferase, which facilitated the maintenance of ROS at optimum levels and decreased O_3_-induced damage (Gupta et al., 2021).

Foliar spraying of catechin and SA can significantly affect the overexpression of *APX* and *CAT* genes, followed by their higher enzymatic activities, leading to lower lipid peroxidation (MDA), and thus increased tolerance of rice to O_3_ (Kittipornkul et al., 2020).

### 6.3 Phytohormone regulation

Similar to ROS and Ca, phytohormones, such as abscisic acid (ABA), SA, jasmonic acid (JA), and ethylene (ET), are involved in the regulation of stomatal aperture movement upon O_3_ exposure, leading to increased plant tolerance, which is mainly related to the cell signaling cascade (Pellegrini et al., 2016). Moreover, O_3_-induced stomatal movement is controlled by anion channels, such as slow anion channel 1 and open stomata 1 (Vahisalu et al., 2010). These channels are regulated by ABA (Negi et al., 2008). However, variations in O_3_ tolerance have been attributed to *g_s_* and other protective mechanisms involved (Castagna and Ranieri, 2009). How phytohormones are associated with plant tolerance to O_3_ exposure needs to be explored to better understand plant responses to stressful conditions. It was reported that the O_3_-mediated increases in JA, asmonoyl-l-isoleucine, and ABA reduce leaf damage in Habataki rice (Tsukahara et al., 2015). Stress-induced apoplastic ROS exacerbates SA synthesis, which contributes to *OsORAP1* expression and causes O_3_ sensitivity and tolerance (Ueda et al., 2015).

Salicylic acid is essential for maintaining antioxidant defense mechanisms and cellular redox responses in plants upon O_3_ exposure (Hasan et al., 2021). Ozone-induced leaf damage accompanying ET biosynthesis has been confirmed by the inhibition of ET biosynthesis in tobacco following O_3_ treatment (Bandurska et al., 2009). This suggests that the suppression of ET biosynthesis can increase O_3_ tolerance in plants. Abscisic acid controls ET and ABA biosynthesis by limiting ABI1 phosphatase activity and ROS homeostasis to induce O_3_ tolerance (Pellegrini et al., 2016). Jasmonic acid is responsible for suppressing ROS-dependent leaf damage under O_3_ stress (Hasan et al., 2021). Ozone exposure induces ET-dependent damage, which can be inhibited by JA when *AT2G24850* and *AT5G24770* are induced by JA (Wang et al., 2017). The significant role of SA in increasing the tolerance of rice to O_3_ was studied by Kittipornkul et al. (2020), where exogenous 100 µM SA supplementation decreased MDA due to higher activity of CAT, APX upon 100–150 ppb (8 h day^−1^) O_3_. Additionally, plants inhibit O_3_ uptake by increasing SA under O_3_ exposure as a mechanism of O_3_ tolerance (Pheomphun et al., 2019). Therefore, a comprehensive research on exogenous phytohormones is required to understand the mechanisms underlying their protective roles in the development of O_3_ tolerance in cultivated crops.

## 7 Mitigation of O_3_ stress in crops

### 7.1 Improving crop management practices

Because abiotic stress is inevitable, it is crucial to develop strategies to combat stress-induced losses in crop production. Agronomic practices, such as changing the cropping season, air quality management, proper irrigation, and adequate plant protection measures, can be used to manage O_3_-induced damage in crops. As O_3_ is strongly linked to seasonal and regional changes, shifting the crop growing season by manipulating the sowing time has been suggested by Teixeira et al. (2011). Seasonal variation in crops can influence the physiological responses of plants by altering their gas exchange capacity, PS I function, and stomatal density upon exposure to O_3_ stress. The generation of adaptive measures has been recorded through decreased *g_s_*, increased stomatal density, and increased PS I activity when plants are exposed to stress at a later stage in their life cycle (Fusaro et al., 2016). Moreover, early sown crops exhibit higher sensitivity to O_3_ owing to their longer life cycles, lengthy post-anthesis stages, higher *g_s,_* and lower threshold levels. In contrast, the comparatively higher enzymatic antioxidant activity of late-grown crops, with increased energy allocation toward growth, facilitates reduced O_3_-induced damage in crops (Yadav et al., 2019; Yadav et al., 2021). Air quality management to check for O_3_ precursors is beneficial during O_3_ exposure. For instance, decreasing methane, an important precursor of O_3_ and greenhouse gases, has proven to be beneficial in combating O_3_ (Shindell et al., 2012). Furthermore, controlling nitrogen oxide emissions in air is beneficial for reducing O_3_ stress in northern China (Lu et al., 2021). By calculating the O_3_ depletion potential of the substances (responsible for O_3_ depletion), Ravishankara et al. (2009) reported that nitrous oxide (N_2_O) is one of the most important greenhouse gases responsible for O_3_ layer depletion. Nitrous oxide production can be mitigated using mulches (e.g., rice straw) and by minimizing fertilizer requirements in the soil. It is evident that soil water-filled pore spaces play an important role in N_2_O emissions in the field; therefore, altering nitrogenous fertilizer with mulch can contribute to N_2_O mitigation (Wu et al., 2018). Intercultural operations, such as mulching, can also reduce O_3_-induced losses.

Although irrigation is desirable for improving crop production, it can enhance the susceptibility of crops to O_3_ toxicity. In irrigated crops, the widely open leaf pores are subjected to stimulated *g_s_* with elevated uptake of O_3_ (Mills et al., 2018). This phenomenon is in agreement with another study (Harmens et al., 2019) that concluded that reduced irrigation could be an effective strategy to mitigate O_3_-induced negative impacts partially or completely by delaying adversities on flag leaves at the time of flower initiation or during the gain-filling period of wheat. Therefore, it is imperative to manage irrigation properly to protect crops from the adverse effects of O_3_ without causing water stress. For example, alternate wetting and drying irrigation was found to be beneficial for increasing rice productivity with decreasing *g_s_* (Carrijo et al., 2017) and was effective in mitigating the adverse impacts of O_3_. In addition, protective measures should be implemented to control plant competition and prevent plant injury in response to O_3_ stress. It has been reported that when weed infestation is coupled with O_3_, it enhances the susceptibility of crops to increased losses compared with O_3_ exposure alone (Li et al., 2016). Ghosh et al. (2020b) reported a higher yield loss in wheat under O_3_ stress owing to weed competition and concluded that strong weed management should be introduced to combat O_3_ stress-affected production loss.

### 7.2 Nutrient management

Although nutrient supplementation is important for enriching soil fertility, improper nutrient maintenance can increase crop vulnerability to various stressors, including O_3_ (Tiwari and Agrawal, 2018). Zhang et al. (2018) conducted an experiment to assess the O_3_ risk management capacity of plant nutrients, where N fertilizer was recorded to elevate the sensitivity of plants to stress; conversely, P improved tolerance by increasing the critical level of O_3_ exposure in crops. Biomass loss caused by O_3_ also differed between the two nutrients. Nitrogen fertilization caused the maximum *g_s_*, which increased the sensitivity to O_3_ and, in turn, resulted in a loss of biomass production, whereas P fertilization decreased *g_s_*. In another study (Tatsumi et al., 2019), it was claimed that a lack of N supplementation in rice caused N deficiency while giving rise to photosynthetic assimilate translocation to the roots in an attempt to increase nutrient uptake, thereby protecting against O_3_-induced damage to plant growth. Nutrient supplementation contributes to detoxification of ROS-induced O_3_. Both N and P were recorded to encourage the mobilization of integrated participation of antioxidant compounds (carotenoid and AsA) and osmoprotectant (proline) and consequently reduce oxidative stress by keeping minimum O_2_^•−^ and H_2_O_2_ while maintaining membrane integrity (Podda et al., 2019). Moreover, N addition can participate in diversifying stored carbohydrates and photosynthates to synthesize amino acids that help repair damage caused by O_3_ (Podda et al., 2019). Although some studies have shown the negative impacts of O_3_ and nutrient interactions, improved plant nutrient management can help mitigate O_3_ stress to some extent (Gautam and Tiwari, 2020).

### 7.3 Carbon dioxide (CO_2_) fertilization

Plant growth can be stimulated by the subsidiary carbon supply provided by CO_2_, which is known as CO_2_ fertilization. This phenomenon can help relieve stress by reducing ROS production during the oxidative damage caused by various stressors, including O_3_ (AbdElgawad et al., 2016). When CO_2_ is used under O_3_ concentrations in chickpeas (*Cicer arietinum* L.), a source-sink imbalance changes with the accumulation of photosynthates in leaves and, subsequently, an alteration of phenological characteristics that ultimately accelerate the crop life cycle to early maturity to escape the damage caused by O_3_ exposure (Singh et al., 2021). Elevated CO_2_ can counteract the damaging effects of O_3_ by increasing shoot biomass and pod weight compared to O_3_-exposed plants alone. Moreover, CO_2_ fertilization can increase protein, starch, and certain mineral nutrients, even under O_3_ exposure, thus combatting the negative impacts of imposed stress (Bhatia et al., 2021). A similar compensation tendency was observed in maize (Yadav et al., 2020). Interactive treatment with CO_2_ and O_3_ resulted in a positive result, as this combination resulted in a higher photosynthesis rate with improved growth attributes and, consequently, an increased yield component compared to the O_3_ treatment alone. Carbon dioxide fertilization also results in improved *g_s_* and carbon assimilation (Yadav et al., 2020). When elevated CO_2_ levels were coupled with elevated O_3_, reduced lipid peroxidation and solute leakage decreased, indicating improved cell membrane integrity. Enhanced antioxidant enzyme activity, which indicates reduced oxidative stress, has also been observed (Kumari et al., 2015). Therefore, CO_2_ fertilization can help mitigate O_3_-induced damage to some extent by reducing O_3_ uptake, increasing carbon assimilation, and reducing oxidative damage, which helps to overcome the detrimental effects on plant growth, physiology (especially photosynthesis), and yield.

### 7.4 Selecting tolerant crop varieties

The use of tolerant cultivars can be an effective strategy that needs to be expanded by including agricultural practices, particularly in O_3_ risk areas (Tiwari and Agrawal, 2018). A noticeable variation in rice genotypes in response to stress was observed by Arshad (2021), in which plant height, dry mass, leaf area, plant damage, and leaf damage were visible between susceptible and tolerant genotypes. Biochemical attributes such as total amino acids, sugar, protein profile, and phenolic content were not affected in varieties tolerant to O_3_ stress, indicating tolerance capacity, whereas a trend of reduction was observed in susceptible genotypes. The susceptible varieties showed early visual symptoms through yellow to brown spots, which later turned into necrosis and early leaf senescence; however, this phenomenon was slower in the tolerant varieties (Arshad, 2021). In wheat, the tolerant variety (HD2967) sensed O_3_ stressors at an early vegetative stage through increased MDA and initiated ROS scavenging activity, and consequently uplifted better antioxidant defense prior to the reproductive stage to protect against yield losses compared to the sensitive genotype (Sonalika), which was later in sensing (Pandey et al., 2018). Moreover, they added that the tolerant variety has tended to translocate more photosynthetic assimilates to increase biomass and husk weight with better resource partitioning, thus ensuring better protection of the reproductive parts under higher levels of O_3_ exposure. These findings agree with those of Dhevagi et al. (2021), who concluded that the tolerant variety of mung beans (*Vigna mungo* L.) had higher AsA under exaggerated O_3_ concentrations, which resulted in better morphological, physiological, biochemical performance, and antioxidant defense than the susceptible variety. Bailey et al. (2019) demonstrated the distinctiveness of a soybean-tolerant variety in the exclusion of O_3_ uptake through stomata by lowering *g_s_* and the transpiration rate, followed by higher water-use efficiency than the sensitive variety. Furthermore, under O_3_ exposure, variations in phosphoenolpyruvate, carboxylase activity, and RuBisCO content and activity controlled the tolerance mechanism of maize hybrids more than the antioxidant defense mechanism of *g_s_*. Stable leaf N content and RuBisCO activity under reduced O_3_ exposure indicated better tolerance to late senescence and better yield than sensitive hybrids (Choquette et al., 2020). Therefore, the selection of tolerant varieties under O_3_ exposure could be an effective approach for adaptation to O_3_.

### 7.5 Using chemical elicitors

Elicitors with different chemical structures can be used against different stressors through exogenous application to crops or incorporation of transcription factors through breeding (Chakraborty et al., 2019). The use of various chemical elicitors to protect against O_3_-induced phytotoxicity has also been demonstrated. For example, chitosan positively influenced wheat growth under O_3_ exposure (Picchi et al., 2021). In addition to improving crop yield and quality, chitosan serves as a protectant that enhances the defense metabolism of plants by increasing the concentration of AsA within a short period by activating the APX enzyme to control H_2_O_2_ and oxidative stress. It also showed limited symptoms on the leaf surface area of chitosan-treated plants compared to O_3_-stressed plants. The same trend of increasing antioxidant enzyme levels under stress conditions was reported by Kittipornkul et al. (2020), using catechins in rice. Application of catechin to rice under O_3_ exposure activated antioxidant enzymes and helped to maintain chlorophyll, *g_s_*, and Mg contents at the vegetative stage, which consecutively resulted in increased panicle number, filled grain weight, and starch, conferring protection against stress. These chemical elicitors protect plants by forming chemical barriers that detoxify O_3_ (Li et al., 2018). It has been shown that trichomes have the capacity to deplete O_3_ near the leaf surface, resulting in reduced O_3_ uptake through stomata (Li et al., 2018). In addition, stress-induced lipoxygenase (LOX) activity (a consequence of O_3_ uptake) were maintained through trichome density, providing plant tolerance to O_3_ exposure. Ethylene diurea is widely used to enhance tolerance to O_3_ exposure, and its effectiveness has been demonstrated in rice (Ashrafuzzaman et al., 2018), maize (Gupta et al., 2020), groundnuts (*Arachis hypogaea* L.; Chaudhary and Rathore, 2020) and castors (*Ricinus communis* L.; Rathore and Chaudhary, 2018). As EDU actively participates in the upregulation of crop growth, photosynthesis, and maintenance of improved membrane properties by activating defense metabolism by increasing AsA and flavonoid content, it can protect against O_3_ stress (Rathore and Chaudhary, 2018; Chaudhary and Rathore, 2020). Furthermore, in addition to improving plant mechanisms (increased SOD, CAT, and APX activities) to regulate the defense system, EDU enhances protein accumulation in plants, which in turn enhances metabolic functions to mitigate damage under O_3_ stress (Gupta et al., 2020). Foliar spraying with calcium acetate and calcium chloride improved tolerance through protectant-induced mechanisms of the antioxidant defense system in O_3_-stressed rice plants (Lakaew et al., 2022). Calcium acetate-treated plants tolerated longer periods of O_3_ exposure by augmenting NAD kinase and NADPH activities. This calcium acetate-mediated increase in NADPH content was associated with higher AsA and GSH levels, and higher APX and GR activities, resulting in an approximately 29% reduction in MDA generation. This calcium acetate-mediated oxidative stress mitigation contributes to improved plant growth and yield (Lakaew et al., 2022).

## 8 Conclusion

Over the past few decades, several studies on the effects of O_3_ on plants have demonstrated that elevated levels of O_3_ hamper overall plant growth and productivity. Ozone can be degraded into ROS in the mesophyll and guard cell walls, which damage the chloroplast ultrastructure and block photosynthetic electron transport after entering directly through the leaves. Leading to stomatal closure and modification of stomatal conductance O_3_ hinder CO_2_ fixation. Ozone induces leaf chlorosis, necrosis, and abscission. Reduced photosynthesis, altered respiration and transpiration, decreased water uptake, disrupted nutrient homeostasis, and the assimilate translocation caused by O_3_ lead to reduced growth. Both chronic and severe O_3_ stress can lead to growth reduction, anomalous reproductive development, yield loss, and crop quality deterioration. Approaches for protecting plant physiological pathways such as photosynthesis, antioxidant defense mechanisms, and hormonal regulation have been reported to enhance plant tolerance to elevated O_3_. Agronomic approaches, such as adjusting planting dates and cropping systems, nutrient management, CO_2_ fertilization, and the use of several chemical stress elicitors have been shown to improve plant performance under elevated O_3_. However, it is difficult to conduct research on ambient O_3_ because it is difficult to measure the amount of ambient O_3_ and the amount of O_3_ entering plants. An appropriate method should be developed to understand these issues, and research on meteorological, biochemical, and physiological aspects should be considered. Literature on the effects of O_3_ on various aspects of plants is readily available, but there is limited availability of literature on strategies for mitigating O_3_-induced stress. Therefore, various agronomic approaches that may mitigate O_3_ stress in plants should be determined. Understanding the biochemistry and physiology of O_3_-stressed plants is vital for developing O_3_-tolerant plants. Therefore, integrated research themes and their implementation are vital for reducing O_3_-induced damage and developing O_3_-tolerant cultivars.

## Author contributions

MH and PV conceived and designed the project; FN, AS, KP, PG, and KN wrote the MS. MH and PV edited the manuscript. All authors contributed to the article and approved the submitted version.
